# Derangement of the Sympathetic Nervous System Following Intermittent Fever

**Published:** 1874-06-15

**Authors:** Dan’l. T. Nelson

**Affiliations:** Mercy Hospital


### DERANGEMENT OF THE SYMPATHETIC NERVOUS SYSTEM FOLLOWING INTERMITTENT FEVER.

CLINIC BY PROF. DAN'L T. NELSON, IN MERCY HOSPITAL, MAY 22, 1874.

PROF. NELSON, in his clinics, this summer, selects a student and assigns him a patient, several days before the clinic, whose case he is to study, as best he can from books or other outside sources, and report the same in detail at the clinic, from written manuscript or otherwise—the class and Professor criticising, or adding to the report, as they may see fit. The following is one of those clinics—J. R. Kewley being reporter for the day:

This young man was attacked, some seventeen months ago, with intermittent fever, or ague of an irregular triple-quotidian variety, previous to which time he had been strong, healthy and robust. After a few months of medication the paroxysms disappeared, and he seemed to be nearly well. At this time, through the advice of a companion, he took a very large dose of cantharides, which was speedily followed by the characteristic symptoms of poisoning by this agent—such as burning along the alimentary canal, with nausea, attempts at vomiting, constriction of throat, and difficult swallowing; pain in the kidneys and along the urinary passages, with violent burning during micturition, etc. He soon seemed to recover from these symptoms, but ever afterward he experienced a languor and dullness, or mental inactivity, at times. He passed but little urine, and that high-colored; tongue heavily coated; appetite, digestion and sleep, good; bowels, at times, regular; again irregular; fæces, of normal color; had no pain any place and, when at rest, seemed quite strong; but upon slight exertion would become much fatigued. He seemed to run on in this way until last July, when his troubles kept him to his bed for some time; since which time his symptoms have corresponded to those before it, with occasionally a chilly feeling in the morning, followed by slight fever in the afternoon. Not improving any, and being unable to attend to business, he sold out and came to the city, in the hope of being cured. After trying quite a number of our city physicians without any beneficial effects, he entered the hospital, nearly three weeks ago. When I first saw him, his symptoms were those already enumerated, with the exception that he has not now any of the chilly mornings, followed by fever in the afternoon. In addition to these symptoms I noticed that, although the pulse was nearly normal in frequency, about eighty, moderately large and full, the artery was quite easily compressed. On auscultation, the heart was found to contract rather quickly (not very strongly), and the interval of rest between the contractions was slightly prolonged. Upon testing the urine I found it normal in quality, except, perhaps, a slightly deficient amount of urea; could not detect any other symptoms, except those of a negative character.

Our text-books, as far as I have examined, give but very little information regarding troubles of this nature.

My preceptor, Prof. N. S. Davis, says, "that in ague that continues to persist for many months, we have the ganglia of the great sympathetic at times affected. Those ganglia, situated in the abdominal region, being first impressed; then the cardiac plexus; and, finally, but rarely, the ganglia within the cranium itself." In these cases the exact nature of the change in the ganglia is not understood, the affection being marked more by the derangement or perversion of the organic functions than by any pathological condition or lesion of these numerous ganglia. We have no pain any place; the patient to his friends may seem well, and even to himself, while in a state of inactivity; but upon exertion of any kind, he finds he becomes wearied; has no strength, no endurance. There may be, as a result of this organic derangement, dullness or stupidity of the brain or mental functions; deficient power in the contractility of the heart, and, as in this case, a rather quick contraction with a prolonged interval of rest; the frequency of the pulse may be normal, but the artery is easily compressed. There may be malnutrition of the body either slight or exaggerated, and, as a result, an anæmic condition of the system; or, upon the other hand, the digestion and assimilation may be fair; appetite and sleep good; the bowels may or may not be regular. I think the derangement or debility of these numerous ganglia will explain many of our patient's symptoms. We can easily understand how this derangement might cause all of the organic manifestations presented by him. We can comprehend how, from this cause, the peristaltic action of the bowels may be diminished or the function of the liver deranged, and thus cause irregularity of the bowels. It will explain the slight derangement of nutrition. We can apprehend how that impaired nutrition of the nerves would depress the muscular tone of the entire system; how, through malnutrition, or sympathy, or both, the encephalon will become more or less deranged and incapable of any great degree of activity. In this patient I would say we have a typical case of this peculiar debility,—or derangement of the sympathetic, following intermittent fever. Probably there was sufficient irritation of the kidneys, resulting from the physiological action of the cantharides on these organs, to account for the small quantity of urine now passed, the patient, at times, micturating but once a day and then only a small quantity. And, as I before stated, urea was somewhat deficient in quantity, in what was voided, indicating a defective elimination of urea from the system. This derangement, we well know, would augment the already pre-existing dullness in the head. I am aware that the treatment the patient is now under, points to the liver as the probable seat of disease in the estimation of his attendants; but I have failed to find sufficient grounds to explain all of the symptoms here presented, by any condition of that organ. It may explain some of the symptoms; but does not the condition of the liver depend, not upon any pathological lesion, but upon the derangement of these organic ganglia, of which I have spoken?

PROGNOSIS.—I do not see any reason why this patient cannot perfectly recover, although it will, undoubtedly, be some time before a cure can be effected. Many months of medication have passed by; but this fact is, I think, accounted for more by incorrect diagnosis, and hence, faulty medication, than by the incurability of the malady.

TREATMENT.—At first thought we might look for tonic remedies, whose action is manifested upon the sympathetic, but as yet we have no remedy that is positively known to act as a tonic on this nervous system. Digitalis does, perhaps, have some such action; it certainly tones up the heart's action. In this case it is strongly indicated, not only for this action, but also on account of its diuretic property. We might add to it some other diuretic, as sweet spirits of nitre. Prof. Davis uses this combination, at times: One part of digitalis to three of sweet spirits of nitre, given in dram-doses about half an hour after meals, properly diluted with water. Strychnia, or nux vomica, with some of the more mild salts of iron, such as lactate, tartrate, or citrate, would undoubtedly be a useful tonic: enriching the blood; toning up the muscular system; in fact, invigorating all the organic functions of the body. It is generally acceded that strychnia acts only on the spinal chord, or at most upon the cerebro-spinal nervous system. Yet Prof. Davis thinks he has seen benefit derived to the sympathetic, from its use, many times. Quinia might be added to the iron and strychnia with benefit, yet there does not seem to be very strong indications for its use now, not so much as there was a few weeks ago. If given at all, it should be used in small tonic doses. The mineral acids might also be used to advantage.

Prof. Nelson—(synopsis)—*Gentlemen:* I think our reporter has given us a fair idea of this case, from beginning to end, and, upon the whole, I agree with him. These cases are not as rare as they used to be, and I would advise you all to give close attention to them. There should be more care and study given to them than to some patients who are stretched upon their backs. The physician is prone to pass them by (and frequently does), with the remark, he is a "hypochondriac," he is "indolent." There is not much the matter; you would be surprised, if you could remove all the disease, to see how small it is! The patient, likewise, would be equally surprised to find himself so much relieved, so much better! The reporter got a wrong idea regarding the condition of the discharges from the bowels, unintentionally on the part of the patient. They are not normal, or, at least, have not been—they being of a brown color instead of a yellowish. The physician is often led astray by the patient saying the fæces are normal, when, if you ask him, "Are they yellow or greenish?" he, not unfrequently, will reply: "No; they are black or brown." In diseases that implicate the alimentary canal I attach more importance to the condition of the discharges, than to the tongue, circulation, etc. First, in these cases, direct your attention to the bowels. If the stools are not normal, you might give some pil. hydg., night and morning. Nitro-muriatic acid with strychnia is very good. We have treated this case thus, giving him also quinia. I do not think the treatment of our reporter can be improved much. To-day we will change the treatment. Digitalis will be given to tone up the circulation and to act as a diuretic; it is one of the best remedies we could use. The kidneys are not much involved in this case I think, there being only, perhaps, a slight irritation remaining from the action of the cantharides. The digitalis will be a sufficient diuretic, I think. The nitro-muriatic acid, strychnia, and quinia will be given three times a day. I think they are the best remedies we could use. In this case, iron is not indicated as much as is generally the case in these patients. I will not give it, although it would do no harm, but probably good. In regard to the action of these medicines: Our books are silent in regard to the tonic action of remedies on the sympathetic; but I think all these remedies, and many others, act directly upon this part of the nervous system. It is only upon this ground that we can explain many of the results of their application in disease. I have no other criticisms or suggestions to make. June 6th, patient is steadily improving.

